# Correction: STAT4 and T-bet control follicular helper T cell development in viral infections

**DOI:** 10.1084/jem.2017045702062018c

**Published:** 2018-03-05

**Authors:** Jason S. Weinstein, Brian J. Laidlaw, Yisi Lu, Jessica K. Wang, Vincent P. Schulz, Ningcheng Li, Edward I. Herman, Susan M. Kaech, Patrick G. Gallagher, Joe Craft

Vol. 215, No. 1, January 2018. https://doi.org/10.1084/jem.20170457

The authors regret that the original version of Fig. 1 (A and C) was published with errors. In A, the percentage of cells in the top right gate of the day 8 FACS plot was corrected to 29.7. In C, the representative numbers for the bottom right Tfh cell gates (IL-21^+^IFN-γ^-^ population) of the FACS plots were inadvertently duplicated from the bottom right Th1 cell gates directly above. These errors do not alter the findings in the published manuscript, as the correct data were used in all calculations and are displayed in the graphs in each figure. The corrected Fig. 1 and corresponding legend appear below.

**Figure 1.**
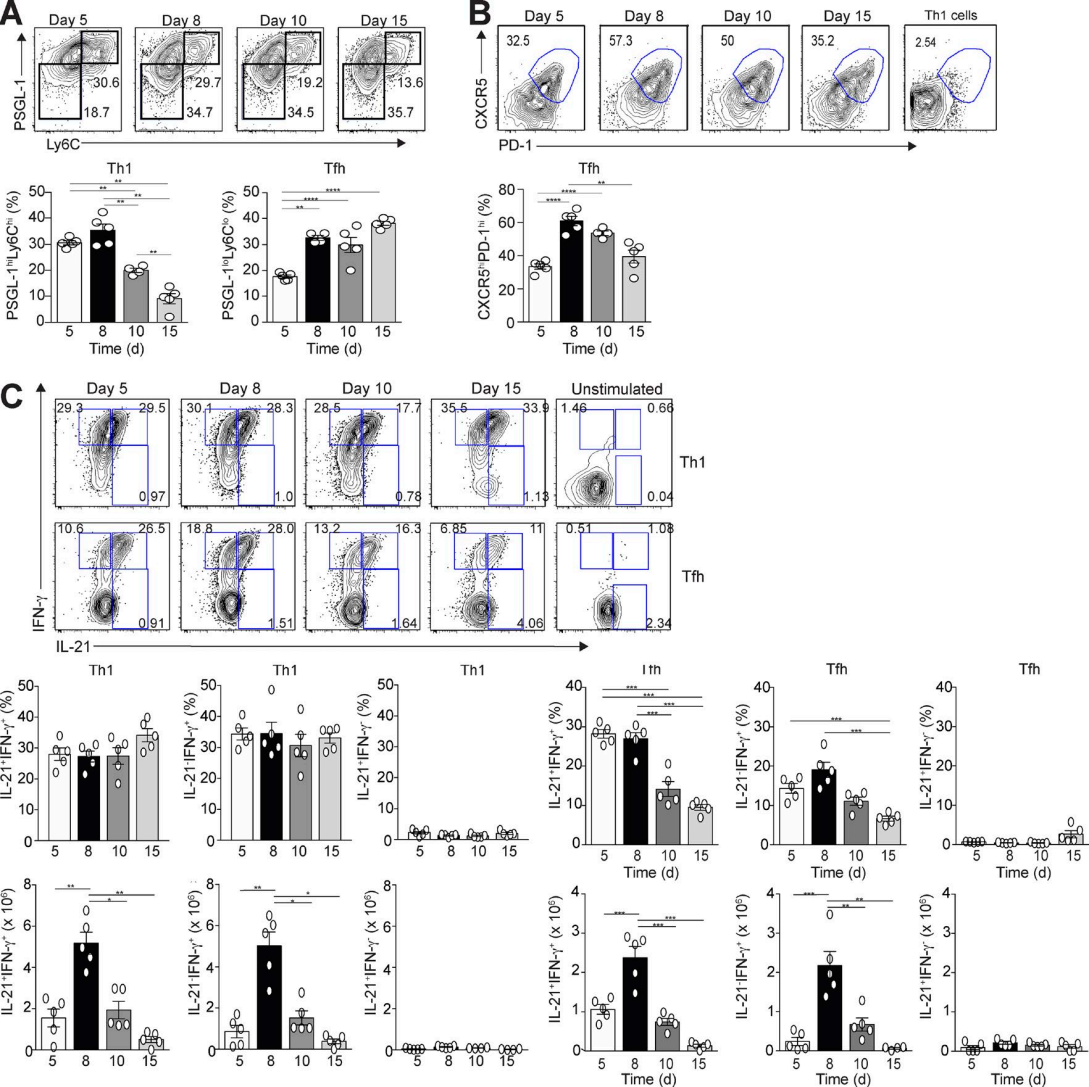
**Temporal production of IFN-γ and IL-21 by Tfh and Th1 cells after acute LCMV infection.** Thy1.1^+^ SMARTA TCR transgenic (Stg) CD4^+^ T cells were transferred into Thy1.2^+^ B6 mice, followed by LCMV Armstrong infection 24 h later. Spleens were harvested at days 5, 8, 10, and 15 p.i. Representative flow cytometry plots of splenic Th1 and Tfh cells, with bar graph summaries, are shown. **(A)** PSGL-1^hi^Ly6C^hi^ Th1 cells or PSGL-1^lo^Ly6C^lo^ Tfh cells with percentages of Th1 and Tfh cells. **(B)** CXCR5 and PD-1 gated PSGL-1^lo^Ly6C^lo^ Tfh cells with cell percentages; gates based on CXCR5 and PD-1 staining of Th1 cells. **(C)** Representative flow cytometry plots of intracellular IL-21 and IFN-γ staining of Th1 and Tfh cells with percentages and numbers of cytokine-positive cells. Data are representative of three experiments with three to five recipients per group. *, P < 0.05; **, P < 0.01; ***, P < 0.001; ****, P < 0.0001 by Student’s *t* test. Error bars represent SEM.

The online HTML and PDF versions of this paper have been corrected. The error remains only in the print version.

